# Age-related hearing impairment and the triad of acquired hearing loss

**DOI:** 10.3389/fncel.2015.00276

**Published:** 2015-07-27

**Authors:** Chao-Hui Yang, Thomas Schrepfer, Jochen Schacht

**Affiliations:** ^1^Department of Otolaryngology, Kresge Hearing Research Institute, University of MichiganAnn Arbor, MI, USA; ^2^Division of Otology, Department of Otolaryngology, Kaohsiung Chang Gung Memorial Hospital, Chang Gung University College of MedicineKaohsiung, Taiwan

**Keywords:** acquired hearing loss, ototoxicity, noise trauma, presbycusis, aminoglycoside antibiotics

## Abstract

Understanding underlying pathological mechanisms is prerequisite for a sensible design of protective therapies against hearing loss. The triad of age-related, noise-generated, and drug-induced hearing loss displays intriguing similarities in some cellular responses of cochlear sensory cells such as a potential involvement of reactive oxygen species (ROS) and apoptotic and necrotic cell death. On the other hand, detailed studies have revealed that molecular pathways are considerably complex and, importantly, it has become clear that pharmacological protection successful against one form of hearing loss will not necessarily protect against another. This review will summarize pathological and pathophysiological features of age-related hearing impairment (ARHI) in human and animal models and address selected aspects of the commonality (or lack thereof) of cellular responses in ARHI to drugs and noise.

## Introduction

Acquired hearing loss is the most common hearing impairment in modern societies. Not all of its causes, however, are of modern origin. Of the major contributing entities, presbycusis or age-related hearing impairment (ARHI) has been recognized by physicians and societies since ancient times. The environmental overload with damaging noise levels, beginning with the industrial revolution, and the introduction of ototoxic antibiotics and anti-cancer agents in the last century added to the spectrum of hazards to the auditory system and established the current triad of drug-induced, noise-induced and age-related hearing losses.

Medical science and research over the last centuries have given us an excellent knowledge of inner ear pathologies and their functional consequences. Recent advances have added insights into molecular pathways, their physiological control mechanisms, and their modulation by environmental and genetic influences. Intriguing biochemical and molecular parallels began to surface, leading to speculation of a common underlying mechanism of acquired hearing loss and the prospect for a unified pharmacological protection. However, details about the individuality of cell death and homeostatic pathways and differentiated responses to attempted protection soon dispelled this notion. This review will focus on ARHI and compare its biochemical and molecular characteristics to ototoxic hair cell death and noise trauma. While the overall pattern of presbycusis is also shaped by changes in central auditory pathways, we will limit our considerations to the cochlea.

## Age-Related Hearing Impairment (ARHI)

### Prevalence

ARHI is the most prevalent form of hearing loss in humans. It is characterized by decreased hearing sensitivity, decreased ability to understand speech in a noisy environment, slowed central processing of acoustic stimuli, and impaired sound localization (Gates and Mills, 2005). Based on a survey from 2001 through 2008, Lin et al. (2011) estimated that 30 million, or 12.7% of U.S. Americans of 12 years and older had bilateral hearing loss with elevated thresholds of >25 dB in the better hearing ear for a speech-frequency pure-tone average at 0.5, 1, 2 and 4-kHz. The percentage of the afflicted population increased dramatically in the elderly. Sixty-three percent of male and 48% of female adults aged between 70 and 79 years were affected. Similar data for Europe indicated that 30% of men and 20% of women had a hearing loss of 30 dB or more at the age of 70 years, and 55% of men and 45% of women at the age of 80 years (Roth, 2015).

First signs of ARHI in males may already be evident at an age of 30–39 years, beginning at the highest frequency range of 10–16 kHz; by 40–49 years hearing impairment has progressed to frequencies of 6–8 kHz. Presbycusis in females generally begins a decade later, and the principal speech band (0.5–4 kHz) is affected in both genders by 60–69 years (Sharashenidze et al., 2007). Besides gender differences, epidemiological studies in the USA have established a higher prevalence in Caucasians than in African-Americans (Lin et al., 2011).

### Genetics

ARHI is shaped by the interplay between genes that govern cochlear integrity and noxious environmental events on the inner ear. Intrinsic factors contributing to presbycusis include genetic disorders rooted in mutations of both nuclear and mitochondrial DNA, hypertension, diabetes mellitus, and other metabolic and systemic diseases (Lee, 2013; Perry et al., 2014). Noise exposure, ototoxic medications, and diet—while being able to cause hearing loss independently—are modulating extrinsic factors of ARHI. The overall impact of individual genetic factors in the development of ARHI in humans remains uncertain but there is evidence for their major contribution in familial aggregation of presbycusis (Raynor et al., 2009). Siblings of individuals with hearing loss had more than four times higher odds of also having hearing loss than siblings of individuals without hearing loss; the risk ratio was greater in women than men. Overall, about half of the variance in this cluster of ARHI could be ascribed to genetic factors the precise nature of which remains elusive. ARHI seems to be polygenic but no major common genetic variants have been identified (Fransen et al., 2015). In agreement with human findings, recent animal studies suggest multiple genetic influences on the progression of ARHI and on the interaction of age with noise in mice (Noben-Trauth and Johnson, 2009; Ohmen et al., 2014). Results from the offspring of 4-way-cross mice provided evidence for several specific alleles that affected hearing at select frequencies, ages, or following noise trauma (Schacht et al., 2012a).

An intriguing question is a potential relationship between presbycusis and late-onset hearing loss due to highly penetrant monogenic mutations. The age of onset of both is fluid but a late-onset hearing loss is generally evident by young adulthood. Any genetic similarities or differences are yet to be determined.

### Impact of Lifestyle and Disease

Lifestyle may influence auditory performance at any age as well as the progression of presbycusis. In a cross-sectional analysis of 164,770 participants, smoking and passive smoking was associated with increased odds of hearing loss in a dose-dependent manner (Dawes et al., 2014). In contrast, moderate use of alcohol was associated with better hearing in the elderly in three independent studies (Popelka et al., 2000; Fransen et al., 2008; Gopinath et al., 2010a), an effect in line with observations that low alcohol intake can be beneficial for general health. Heavy drinkers, in contrast, as well as those with a high body mass index, had a tendency toward more pronounced high-frequency hearing loss. Such differential influences of lifestyle might be related to overall health, especially in association with cardiovascular disease. A cause of microvascular abnormalities that also might affect hearing is type 2 diabetes mellitus (Mitchell et al., 2009). Type 2 diabetes, often associated with overweight and obese body habitus in adults as well as in youth, is a common metabolic disease that causes impairment of various organ systems. Data from a large longitudinal cohort study indicate that type 2 diabetes is associated with prevalent hearing loss and a significant accelerated progression of hearing loss in patients with newly diagnosed diabetes (Mitchell et al., 2009).

Since diet is one of the few potentially modifiable risk factors for age-related hearing loss, the impact of nutritional behavior has frequently been investigated (Gopinath et al., 2010a, 2011b). Both a high-carbohydrate diet and a high dietary intake of cholesterol were associated with ARHI. In contrast, treatment with 3-hydroxy-3-methyl-glutaryl (HMG) CoA reductase drugs (statins) and consumption of monounsaturated fats and cereal fibers inclined toward a lower prevalence of hearing loss. We will discuss more aspects of dietary manipulations of ARHI in the “Antioxidants and Vitamins in Human Hearing and Prevention of Hearing Loss” Section.

## Cochlear Pathology in Humans and Animal Models

A hallmark of human ARHI is the variability of its pathology. Although high-frequency hearing loss appears to be a prevalent form, some individuals may also show a gradually sloping or a flat audiometric curve (Suga and Lindsay, 1976). Moreover, different structural elements can be affected such as hair cells, neurons, lateral wall tissues, or a combination thereof (Nelson and Hinojosa, 2006). This variability has been ascribed to genetic susceptibility modified by environmental effects of noise exposure, disease, and lifestyle. In practice, ARHI is largely an umbrella term for multiple forms of auditory pathology manifest in aging individuals.

In contrast, drug- and noise-induced hearing loss tend to present a well-defined pattern of damage in both human and experimental animals. Both aminoglycoside antibiotics and cisplatin primarily damage outer hair cells in a predictable base-to-apex progression. Noise trauma may initially affect fibrocytes of the spiral ligament (Hirose and Liberman, 2003) and will—at higher intensities or longer duration—damage hair cells in relation to the frequency and intensity of the stimulus.

### Sensorineural Elements

Sensory hair cell loss has been documented in temporal bones of patients with ARHI but does not appear to be its only determining cause. The area of destruction was usually located in the basal end of the cochlea causing a threshold elevation at 8 kHz but rarely extended towards the apex far enough to involve speech frequencies (Schuknecht and Gacek, 1993). More recent studies of temporal bones have expanded this view by showing significant degeneration of multiple cochlear elements including stria vascularis and spiral ganglion cells that better correlate with the severity of hearing loss (Nelson and Hinojosa, 2006). Specifically, the extent of ganglion cell degeneration was associated with the slope of the descending high-frequency component of the audiogram. In addition, several reports have indicated nerve damage in the presence of apparently intact hair cells in ARHI (Sone et al., 1998; van Ruijven et al., 2005; Linthicum and Fayad, 2009).

Animal models that reflect a sensorineural ARHI with loss of outer and, later, inner hair cells include aging rats and mice. CBA/J mice as well as the CBA/CaJ and CAST strains present little evidence of hearing loss until 18 months of age, i.e., long into their lifespan, when they begin to show hair cell loss and elevated auditory thresholds (Li and Borg, 1991; Sha et al., 2008). In contrast, the C57BL/6, BALB and DBA/J strains develop a severe high-frequency sensorineural hearing loss as early as 5 weeks in DBA or 3 months of age in C57BL/6 (Zheng et al., 1999). Those strains carry a mutation of the cadherin 23 (*Cdh23*) gene at the *ahl* locus predisposing them to accelerated hearing loss, distinct from the late-life age-related changes in other mouse strains and in human. Indeed, no convincing evidence yet exists associating variants in human *CDH23* with ARHI. For example, a recent study of 310 aging participants with poor hearing vs. 308 participants with good hearing found no association of a common *CDH23* variant with presbycusis (Hwang et al., 2012b). These facts should therefore disqualify strains carrying the *Cdh23*^ahl^ allele as models of typical ARHI in human. Likewise, mouse models with defective mitochondrial DNA repair mechanisms may show an accelerated hearing loss that does not reflect human presbycusis (see “Involvement of Mitochondria” Section).

Degeneration of spiral ganglion cells can also be observed in animal models of aging. The degeneration may follow the loss of hair cells but may also occur independently (Keithley et al., 1989; Sha et al., 2008). Conversely, spiral ganglion cells may be preserved in the presence of a degenerated cochlea (White et al., 2000). More recently there has been evidence for synaptopathy as a pathophysiological process underlying supra-threshold deficits. In mice, degeneration of cochlear synapses in age-related hearing loss preceded both cochlear nerve degeneration and hair cell loss and occurred long before a threshold elevation was measurable (Kujawa and Liberman, 2015). In fact, these observations may relate to a report by Crowe et al. (1993) on “dendritic presbycusis” in which human temporal bones presented a normal cochlea but a loss of dendritic processes of the spiral ganglion (Rizk and Linthicum, 2012).

Synaptopathy may also be a consequence of noise exposure or aminoglycoside treatment. Following a noise exposure regimen that initially only led to a temporary threshold shift as assessed by auditory brainstem response (ABR), compound action potential and distortion-product otoacoustic emission (DPOAE; Kujawa and Liberman, 2009), the synapses from the afferent nerve fibers to the inner hair cells were the most vulnerable elements at a later age, not the hair cells, resulting in an attenuation of supra-threshold neural responses. Threshold elevations without a reduction in hair cell counts have also been observed after kanamycin administration in guinea pigs (Nicol et al., 1992). In a mouse model of enhanced endoplasmic reticulum stress (Oishi et al., 2015) gentamicin caused synaptopathy; spiral ganglion cells and inner hair cell synapses were reduced while hair cells remained intact by morphology and DPOAE measurements.

The complexity of age-related changes clearly calls for carefully selected animal models and detailed assessments of auditory pathophysiology. Appropriate animal models would develop hearing loss with advanced age and not be confounded by mutations that disrupt the physiological aging process. In addition to loss of hair cells, neuronal elements including synaptic connections need to be evaluated. As a physiological measure, ABR thresholds are insufficient just as a pure-tone audiogram would be insensitive to diffuse neural degeneration and an impaired understanding of complex sounds in humans. Therefore animal experimentation needs to consider supra-threshold deficits and performance in simple behavioral tasks such as gap detection (Altschuler et al., 2015).

### Stria Vascularis and Spiral Ligament

As briefly mentioned in the preceding section, age also changes the morphology of cochlear supporting tissues. For example, the volume of stria vascularis and spiral ligament was significantly lower in temporal bones of older (64–84 years old) than in younger (15–38 years old) subjects (Ishiyama et al., 2007). More to the point, strial atrophy was a frequent occurrence in temporal bones of individuals that had been diagnosed with ARHI (Schuknecht et al., 1974; Suga and Lindsay, 1976; Nelson and Hinojosa, 2006). However, there is no indication that strial pathology can be a sole cause of hearing loss. In fact, the best correlation between the severity of ARHI and morphological changes was established for the extent of degeneration of all four cochlear elements, namely stria vascularis, spiral ganglion cell, inner and outer hair cells (Nelson and Hinojosa, 2006). The observation that ABR thresholds were not associated with a single peripheral deficit was also made from a study of age-related hearing loss in rhesus monkeys (Engle et al., 2013). Spiral ganglion density and loss of outer hair cells contributed to the audiometric impairments but neither did decreases in inner hair cell numbers or thickness of stria vascularis. The strongest correlation was seen with the number of different pathologies present.

Human studies cannot evaluate the metabolic consequences of strial pathology on the endocochlear potential. Animal models indicate strial dysfunction is not a prerequisite for loss of hair cells. Although CBA/CaJ mice may display a gradual decline in endocochlear potential during aging, other strains such as CBA/J maintain a stable potential (Sha et al., 2008; Ohlemiller et al., 2010) while developing ARHI. Conversely, however, a significant decline in endocochlear potential should lead to impaired auditory processing. Among animals, the Mongolian gerbil is considered a model for a strial contribution to hearing loss with age. Strial atrophy was variable but Na, K-ATPase activity and the endocochlear potential tended to decrease (Schulte and Schmiedt, 1992).

Morphological changes in the stria may also be associated with treatment by drugs and noise but may not be causal to hearing loss. Strial pathology can be observed after aminoglycoside challenge (Forge et al., 1987), but the endocochlear potential remains stable until late in ototoxic treatment (Komune et al., 1987; Xiong et al., 2011), negating a functional consequence of such alterations. Cisplatin may affect both strial morphology and function (Laurell et al., 2007) but at least some of these changes are reversible (Klis et al., 2000) and therefore not a direct cause of hair cell loss. The effects of noise on lateral wall tissues are variable, probably determined by the intensity of the treatment or species differences, but noise trauma caused degeneration of fibrocytes and edema with temporary changes to the endocochlear potential (Hirose and Liberman, 2003). Considering the sum of the evidence it appears that strial pathology, although seen to some extent in the aging and drug- or noise-treated cochlea, is not a primary or decisive cause of acquired hearing loss.

## Molecular Pathology

### Oxidative Stress

Potentially detrimental reactive oxygen species (ROS) are produced in every cell by enzymatic reactions and mitochondrial respiratory mechanisms. However, only their excess—either via overproduction or insufficient detoxification—will create oxidative stress and potentially trigger cell death. A well-maintained redox balance is important for the cochlea as for any other tissue. Transgenic mice with deficiencies in antioxidant systems, lacking the enzyme copper/zinc superoxide dismutase, lost hair cells and auditory neurons to a greater extent than their normal littermates (McFadden et al., 1999; Keithley et al., 2005). Outer hair cells, especially those in the base of cochlea, appear to be highly sensitive to ROS damage as compared to supporting cells (Sha et al., 2001).

Human studies have only been able to approach a potential link between oxidative stress and hearing performance indirectly via the determination of stress markers in serum. A group of 63 subjects with diabetes mellitus and diabetic hearing impairment showed significant increases in serum levels of oxidation markers and enzymes such as glutathione peroxidase and superoxide dismutase (Aladag et al., 2009). Serum levels of several ROS species were also determined in a cross-sectional study on 302 subjects aged 40–77 years with or without clinically diagnosed age-related sensorineural hearing loss (Hwang et al., 2012a). Assays reflecting the combined concentrations of hydrogen peroxide, hypochloride and hydroxyl radicals showed a significant positive association between their elevated levels and pure-tone thresholds at low (250–1,000 Hz) and high frequencies (2,000–8,000 Hz). An indicator for superoxide, in contrast, only implied an association with low-frequency thresholds. While such associations are intriguing they cannot provide information on potential causal relationships or the redox status of the auditory system. Interestingly, the use of antioxidant supplementation (vitamins C and E, folate, coffee, and tea), which was reported by 66% of the participants in the latter study, had no influence on auditory performance.

Animal studies, unfortunately, do not resolve the question of how oxidative stress relates to the development of ARHI. In a CBA mouse model of age-related hearing loss (Jiang et al., 2007) markers of oxidation began to appear at 12–18 months of age in the organ of Corti and later (at 23 months) in spiral ganglion cells, stria vascularis and spiral ligament. Conversely, components of cellular antioxidant systems had decreased by 18 months in the organ of Corti. This evidence of an imbalanced redox system resembles the pattern of oxidative stress in animal models of drug-induced hearing loss and noise trauma. A striking contrast, however, is the fact that a case can be made for a causal relationship in the latter two pathologies but not in ARHI: dietary manipulations reducing oxidative stress effectively protect against aminoglycoside ototoxicity and the permanent effects of noise trauma but not against AHRI (Oishi and Schacht, 2011; Xie et al., 2011).

The complexity of mechanisms underlying age-related cochlear pathology and pathophysiology were further illustrated by studies employing caloric restriction in mice. Caloric restriction is a well-established regimen to increase stress-resistance by curbing ROS (Sohal and Weindruch, 1996). Such a diet can extend longevity, slow age-related physiological declines, and decrease tumor and disease burden in a variety of animal species. Willott et al. (1995) evaluated ARHI in 15 different mouse strains maintained on a well-controlled calorically restricted diet beginning at a young age. The results varied profoundly between strains. They ranged from an amelioration of age-related hearing loss (as measured by auditory brainstem response or cochlear pathology) to no apparent effect and to an acceleration and exacerbation of hearing loss. Hence, genetic heterogeneity had a greater influence on auditory performance than caloric restriction. By extension, genetics can be expected also to influence the outcome of dietary manipulations with antioxidants.

From the sum of these results, we can postulate that oxidative stress can accompany but is not an obligatory condition for ARHI. The “Antioxidant Protection in Animal Models” Section will further explore this notion.

### Involvement of Mitochondria

A decisive role of mitochondria in the aging process in general seems firmly established. Mitochondria are particularly vulnerable to the cumulative effects of genetic or environmental damage because mitochondria, in contrast to nuclei, lack an efficient DNA-repair system and protective histones. Hence, mutations in the mitochondrial DNA (mtDNA) for the 13 proteins that it encodes are more likely to occur and persist in a lifetime than mutations in nuclear DNA. As a consequence, mitochondrial function may be compromised and pathology can become evident, varying with cell type and the type of mutation (Lee and Wei, 2012). While much emphasis has been placed on the formation of ROS by mitochondria, recent evidence expands the spectrum of mitochondrial dysfunction to their turnover (fusion and fission) and energetics, regulation of calcium dynamics and apoptosis (Gonzalez-Freire et al., 2015).

An involvement of mitochondria in ARHI is supported by several lines of evidence. Patterns of maternal inheritance of presbycusis are one indication (Hutchin and Cortopassi, 2000). Likewise, a considerable literature confirms mitochondrial mutations in the temporal bones of patients with presbycusis (Fischel-Ghodsian et al., 1997). Such mtDNA mutations are highly variable, targeting different segments of the DNA, and often involving large-scale deletions in genes necessary for energy metabolism (Chen and Tang, 2014). Specifically, a mtDNA4977 deletion was more prevalent in temporal bones from patients with presbycusis than in individuals with normal audiograms (Bai et al., 1997) or in age-matched controls and young and middle-aged groups (Dai et al., 2004). A comparison of individual audiometric thresholds with levels of the mtDNA4977 deletion in archival human cochlear tissue (quantified by polymerase chain reaction) was able to establish a significant correlation between the severity of presbycusis and the deletion (Markaryan et al., 2009). While this evidence is solid, another study indicated that mitochondrial mutations *per se* do not determine the rate of age-related deterioration of hearing. An analysis of the mitochondrial genome of 200 individuals with normal hearing and 200 with significantly elevated thresholds (Bonneux et al., 2011) found a lack of an association between the mutation load of inherited mitochondrial variants and ARHI. A combination of mitochondrial defects with other environmental or genetic influences on the inner ear has to be postulated in these cases.

As animal models reflecting mitochondrial pathology, mouse mutants deficient in mitochondrial proofreading have been suggested (Kujoth et al., 2005). However, these animals are afflicted with dramatic degenerative changes in phenotype with devastating effects on the animals’ health that are not shared by late-life wild-type mice. Such “catastrophic mutations” do not represent a normal aging process (Gershon, 2005; Miller, 2005) and we have to treat results from these mouse strains with caution. Another example of a confounding mitochondrial mutation is the DBA/2 mouse which, in addition to harboring the *Cdh23^ahl^* allel, presents defects in the respiratory chain (Someya et al., 2007; Johnson et al., 2008). It is yet to be established whether information derived from these models contributes to or distracts from our understanding of ARHI.

Among the other forms of acquired hearing loss, a direct effect on mitochondria can be deduced for aminoglycoside ototoxicity: the decoding site of mitochondrial RNA is structurally highly similar to the corresponding decoding site in bacteria, where aminoglycosides bind and inhibit protein synthesis. Drug affinity to mitochondrial RNA has indeed been correlated with ototoxicity (Shulman et al., 2014) and non-ototoxic aminoglycosides have been developed on the basis of differential binding to bacterial and mitochondrial RNA (Duscha et al., 2014). As a consequence (or independently) the drugs may disrupt the respiratory complexes (Jensen-Smith et al., 2012) compromising energy metabolism or initiating cell death pathways.

The mechanism by which mitochondria might participate in acute noise damage may have several components. A reduction in cochlear blood flow and oxygen availability would lead to inefficient oxidative phosphorylation and a rapid depletion of ATP levels (Chen et al., 2012). This impairment of mitochondrial function may be coupled with a “reperfusion effect” when blood flow resumes, causing excess ROS formation. An elevation of intracellular free calcium following noise exposure (Fridberger et al., 1998) is another potential trigger of mitochondrial dysfunction. Although calcium does not directly affect the respiratory chain, it may affect mitochondrial membrane permeability in addition to activating cell-damaging lipid peroxidation or protein kinase pathways.

In summary, there is clear evidence that mitochondria play a role in all three forms of acquired hearing loss (Böttger and Schacht, 2013). However, the detailed mechanisms diverge between these pathologies, perhaps even with only little overlap. Primary disruptions appear to be mtDNA deletions (in ARHI), dysfunction of mtRNA processes (aminoglycosides), and a compromised respiratory metabolism (noise trauma), providing examples of the multifaceted contributions of a single organelle.

### Cell Death Pathways in Animal Models

Following a lethal insult, cells will undergo a controlled demise. The classical forms of cell death, apoptosis and necrosis, and related pathways have been documented for ARHI in animal models where they may operate in parallel or sequentially. Apoptosis was evident in aging rat cochleae and in outer hair cells of both middle-aged (12-month-old) and old (18–26-month-old) CBA/J mice with hearing deficits. The middle-aged mice also bore nuclei with necrotic features, which were absent from old mice (Hu et al., 2008; Sha et al., 2009).

Essential effectors of intrinsic apoptosis are the caspases, proteases that are sometimes termed “executioner” proteins. Their activities are modulated by the Bcl-2 family of apoptosis regulators. Bax and Bak, pro-apoptotic proteins in the Bcl-2 family, translocate to mitochondria and lead to an increase of mitochondrial membrane permeability resulting in the release of cytochrome c; activated caspase-9 and caspase-3 then initiate signaling pathways towards cell death. These caspase-dependent pathways and the participation of Bcl family of proteins have been established for age-related hearing loss *in vivo* in the gerbil, the rat, and the mouse (Alam et al., 2001; Hu et al., 2008; Sha et al., 2009). Commensurate with apoptotic events is the translocation of endonuclease G in animal models of ARHI (Sha et al., 2009). Apoptosis-inducing factor and endonuclease G translocate from mitochondria to the nucleus, promoting chromatin condensation and DNA fragmentation.

Another route to cell death associated with ARHI is the MAPK pathway. Mitogen-activated protein kinases (MAPKs) are a family of kinases that include c-jun NH2-terminal kinases (JNK), p38 MAPKs, and other extracellular signal-regulated kinases. Immunohistochemical analyses in the cochlea of aging CBA/J mice demonstrated increased phosphorylation (i.e., activation) of JNK and p38 MAPK in outer hair cells (Sha et al., 2009). A screen for gene expression patterns in the aging CBA mouse cochlea (Tadros et al., 2008) likewise found significant changes with age that included the family of caspases, MAP kinases, and calpains.

Calpains and cathepsins are calcium-dependent proteases that activate downstream pathways by proteolysis of target proteins. They are components of caspase-independent signaling to cell death, released from lysosomes in response to increased intracellular calcium, and implicated in apoptotic and necrotic cell death. Both of these proteases are activated in the aging mouse cochlea (Sha et al., 2009). In addition, the same study also demonstrated cytochrome c release, activation of caspase-9, translocation of endonuclease G and the involvement of the MAPK pathway, indicating that multiple cell death pathways can be proceeding concurrently.

There is considerable overlap in cell death pathways between the three pathologies. Apoptotic markers have been demonstrated for aminoglycoside ototoxicity in guinea pig cochlear and vestibular sensory epithelia (Lang and Liu, 1997; Nakagawa et al., 1998), for cisplatin ototoxicity in the gerbil (Alam et al., 2000), and for noise-induced hearing loss in the guinea pig and chinchilla (Yang et al., 2004). Necrotic hair cells appeared in response to aminoglycosides in CBA/J mice (Jiang et al., 2006a) and following noise exposure in the chinchilla, but have rarely been observed as a result of cisplatin ototoxicity. Active caspase-3 and caspase-9 or cytochrome c release may accompany hair cell death in response to aminoglycosides in various model systems (Cunningham et al., 2002; Wei et al., 2005), to cisplatin *in vivo* (Devarajan et al., 2002), and following noise exposure (Nicotera et al., 2003). Upstream of caspases, Bcl-2 family proteins were induced by aminoglycosides in the zebra fish lateral line (Coffin et al., 2013), by cisplatin in the gerbil, and by noise in the CBA/J mouse and the guinea pig (Vicente-Torres and Schacht, 2006; Yamashita et al., 2008). Translocation of endonuclease G is also evident in animal models of aminoglycoside ototoxicity (Jiang et al., 2006a) and noise-induced hearing loss (Yamashita et al., 2004). Further extending the similarities, p38 MAPK and JNK are involved in aminoglycoside ototoxicity (Pirvola et al., 2000; Wei et al., 2005) and noise trauma (Wang et al., 2003; Jamesdaniel et al., 2011). Finally, both calpain and cathepsin were activated in response to aminoglycoside treatment *in vivo* (Jiang et al., 2006a) and calpain in response to noise exposure (Wang et al., 1999).

This commonality of cellular responses reflects the fact that even widely different initial insults to cell integrity will converge into a few final pathways of cell death. Intimately linked to the emergence of such pathways is the recruitment of survival mechanisms. The balance between these competing forces will ultimately decide cell fate.

## Protection from Cochlear Pathology and Pathophysiology

### Homeostatic (Cell Rescue) Pathways in Animal Models

A first response of cells under attack by potentially injurious stimuli will be to maintain their integrity by evoking homeostatic pathways. In the case of acquired hearing loss, upregulation of antioxidant defenses appears to be a ubiquitous response. In fact, in a direct comparison, Chen et al. (2013) demonstrated the upregulation of the antioxidant peroxiredoxin 3 in mice *in vivo* after acute noise exposure, after a 2 week treatment with kanamycin, and in the cochlea of 19-month-old animals. Likewise, heat shock proteins which stabilize protein structure against cell stress will attenuate loss of hair cells or auditory function by drugs (Taleb et al., 2009), noise (Yoshida et al., 1999), and age (Mikuriya et al., 2008). Another canonical protective pathway mediated by phosphatidylinositol 3,4,5-trisphosphate/Akt signaling has been documented for aminoglycoside ototoxicity (Chung et al., 2006; Jiang et al., 2006b) and ARHI (Sha et al., 2010). Both the knowledge of cell death and molecular rescue pathways can provide a rational basis for the choice of pharmacological agents to prevent or attenuate acquired hearing loss.

### Antioxidant Protection in Animal Models

There was early tantalizing support for antioxidant protection against ARHI. A prospective randomized study had suggested that dietary supplementation might delay or mitigate auditory damage. Treated Fischer 344 rats had improved auditory sensitivities in old age but the improvements were small and differed with different antioxidants while the number of long-term surviving animals was low (Seidman, 2000). Later studies using a variety of different approaches yielded ambiguous results or were met with failure, shedding doubt on the general applicability of such an intervention.

The deterioration of auditory neurons and stria vascularis was reduced in beagle dogs that were fed an antioxidant-enriched diet (supplemented with vitamin E, l-carnitine, lipoic acid, and vitamin C) for the last 3 years of their lives (Le and Keithley, 2007). Unfortunately, the study was confounded by excessive noise in the dog kennel and it could not be ruled out that some of the pathology attenuated by the diet had been noise-induced. The same authors investigated transgenic mice overexpressing mitochondrial superoxide dismutase (SOD2) as a means to reduce oxidative stress. Contrary to expectation, hearing loss at 20 months of age was greater in transgenics than in the parent strain of B6 mice. This result complemented an earlier study (Keithley et al., 2005) in which the overexpression of cytosolic copper-zinc superoxide dismutase (SOD1) also did not prevent age-related hearing loss.

Outright failures of dietary intervention were observed in an attempt to lower oxidative stress by strengthening mitochondrial energy metabolism with acetyl L-carnitine in 15- to 24-months old rats (Bielefeld et al., 2008). Auditory performance was not affected although it could be argued that the duration of intervention of up to 90 days might have been insufficient. Sha et al. (2012) subjected CBA/J mice to a long-term dietary treatment from 10 to 22 months of age, spanning a period from normal auditory thresholds to significant hearing loss. Food enriched with a combination of vitamins A, C, E and α-lipoic acid did not delay or attenuate age-related hearing loss.

In striking contrast to ARHI, reliably positive results have been achieved with antioxidants in preventing hearing loss caused by drugs or noise. Recent review articles have extensively discussed mechanisms and therapeutic approaches to reduce the ototoxic impact of cisplatin and aminoglycosides (Huth et al., 2011; Xie et al., 2011; Schacht et al., 2012b) as well as noise trauma (Oishi and Schacht, 2011). This contrast in efficacious protection reinforces the conclusion put forth in the “Oxidative Stress” Section that oxidative stress can accompany ARHI but cannot its sole cause.

### Antioxidants and Vitamins in Human Hearing and Prevention of Hearing Loss

Lifestyle and diet influence both our general health and our sense of hearing (see “Impact of Lifestyle and Disease” Section). To what extend these two effects can be distinguished is an interesting theoretical question but of no practical consequence. Diet is an easily modifiable factor and any delay in ARHI would have important implications for public health. Unfortunately, there is no simple remedy.

#### Influence of Vitamins on the Prevalence of Hearing Loss

An adequate intake of vitamins is essential for normal auditory function as a number of cross-sectional studies have demonstrated. Spankovich et al. (2011) compared pure-tone audiometry and otoacoustic emissions with dietary data for 2,111 adults, aged 49–99 years of age. Better hearing was associated with higher reported intake of vitamin C, vitamin E, riboflavin (vitamin B_2_), magnesium, and lycopene (a carotenoid antioxidant). Similarly, an epidemiological study of 1,910 Korean subjects aged 50–80 years compared their hearing thresholds with self-reported intake of dietary supplements (Kang et al., 2014). The intake of vitamin C, and to a lesser extent retinol (a vitamin A analog), riboflavin (vitamin B_2_), and niacin (vitamin B_3_) positively correlated with better thresholds. A survey of a general population in the USA aged 20–69 years (2,592 subjects) likewise found that diets high in vitamin C, beta-carotene, and magnesium were correlated with better hearing (Choi et al., 2014).

However, results from surveys of different populations do not always agree. For example, auditory thresholds were impaired in subjects (55 women aged 60–71 years) with low serum levels of folic acid (vitamin B_9_) and vitamin B_12_ (Houston et al., 1999). Another cross-sectional study of presbycusic subjects confirmed that low serum folate levels (<11 nmol/L) increased the odds of prevalent mild hearing loss (Gopinath et al., 2010b). In contrast, Berner et al. (2000) failed to demonstrate any association between hearing level and vitamin B_12_ or folic acid in 91 elderly subjects. A poor vitamin B_12_ status was also evident in 93 older adults with hearing loss (Park et al., 2006) but short-term dietary supplementation with vitamin B_12_ did not affect their hearing.

Another caveat is that an overuse of vitamins may be detrimental to the auditory system. Higher vitamin A intake (Spankovich et al., 2011) and higher serum levels of vitamin D (Kang et al., 2014) have been associated with worse hearing.

#### Influence of Vitamins on the Incidence of Hearing Loss

Even if a balanced or fortified diet generally promotes good health and hearing, a direct impact of nutritional supplements on the progression of ARHI remains debatable. A good example of a discord between the influence of nutrition on prevalence and on incidence of hearing loss comes from cross-sectional and longitudinal analyses of 2,956 participants of age 50 and older (Gopinath et al., 2011a). A segment of the study group that took dietary vitamin E or vitamin A had a 14% and a 47% reduced risk, respectively, of prevalence of a moderate (or greater) hearing loss. Over a 5-year follow-up period, however, an increased dietary intake had no influence on the incidence of ARHI during that time.

A prospective cohort study of 26,273 men aged 40–74 years reported a similar lack of benefits (Shargorodsky et al., 2010). The group was followed for 18 years and 3,559 cases of hearing loss were documented. There was no significant association between the risk of hearing loss and the self-reported intake of Vitamin B12, vitamin C, vitamin E, or beta carotene.

The utility of antioxidant treatments was further cast in doubt in a recent prospective, placebo-controlled, double-blind and randomized trial with 120 participants of 60 years of age or older (Polanski and Cruz, 2013). The subjects were treated for 6 months with one of four regimens: α-lipoic acid plus vitamin C, papaverine chlorhydrate plus vitamin E, gingko extract, or placebo. The various supplements had no effect on pure tone audiometry, speech recognition threshold, or percentage index of speech recognition, regardless whether measured between groups or longitudinally over time.

A possible exception to this scenario of failures is folic acid. A double-blind randomized and placebo-controlled study in 728 older men and women (Durga et al., 2007) provided evidence for the efficacy of folic acid supplementation. Over a 3-year period, the decline in low-frequency hearing was significantly attenuated in elderly participants compared with a placebo group. However, the effect was modest (0.7 dB) and high-frequency hearing was not rescued. A caveat applies: the study was conducted in the Netherlands where food products are not fortified with folic acid. In many industrialized countries, folic acid is added to flour in order to prevented neural tube defects in infants. However, confirmative data were obtained in the prospective cohort study cited above (Shargorodsky et al., 2010). Folate reduced the risk of hearing loss in men older than 60 years when comparing the highest quintile vs. lowest quintile of intake. The authors offer the explanation that high folic acid intake is necessary to overcome an age-related increased prevalence of folate malabsorption and folate depletion.

#### Antioxidants as Protection Against Drugs and Noise

The reliable protection achieved with antioxidants against drugs and noise trauma in animal models has prompted several trials in humans. Based on the efficacy of salicylate in a guinea pig model of ototoxicity (Sha and Schacht, 1999), aspirin (acetyl salicylate) was co-administered with gentamicin in a randomized double-blind clinical trial involving 195 patients (Sha et al., 2006). Fourteen of 106 subjects in the control group but only 3 of 89 in the aspirin group met the criteria of hearing loss (≥15 dB threshold shift at both 6 and 8 kHz unilaterally or bilaterally), representing a 75% reduction of the incidence of hearing loss. This study provided the first proof-of-principle that it is possible to translate animal experiments in the field of acquired hearing loss to the clinic. An independent follow-up trial with 30 patients per group confirmed the efficacy of aspirin (Behnoud et al., 2009). Another antioxidant, N-acetylcysteine (NAC) also attenuated ototoxicity caused by aminoglycosides in patients with bacteremia receiving hemodialysis (Feldman et al., 2007). Of 20 patients in the control group, 11 showed elevated pure-tone hearing thresholds at the follow-up examination as opposed to only 2 of 20 patients in the NAC group. In contrast, a small-scale study of vitamin E in the prevention of aminoglycoside ototoxicity was not successful (Kharkheli et al., 2007).

Therapeutic agents to mitigate noise-induced hearing loss in human subjects have included magnesium which is primarily a vasodilator, not an antioxidant (Attias et al., 1994), and NAC. In an attempt to reduce temporary threshold shifts after exposure to loud music in 31 normal subjects, NAC was not effective in protecting pure-tone thresholds and distortion product otoacoustic emissions (Kramer et al., 2006). Likewise, Lindblad et al. (2011) found no significant differences in temporary noise-induced threshold shifts and DPOAE amplitude in military personnel after a shooting exercise. However, more sensitive supra-threshold measurements (psycho-acoustical modulation transfer functions) were compromised in 23 noise-exposed controls but not in 11 subjects receiving NAC. Another double-blind cross-over study assessed the efficacy of NAC in 53 male Taiwanese steel workers that were also genotyped for deficiencies in glutathione S-transferases, enzymes that protect against oxidative stress (Lin et al., 2010). Noise-induced temporary threshold shifts were small, averaging ~3 dB at high frequencies (3,000–6,000 Hz). NAC reduced the threshold shifts only in a subset of workers with specific null genotypes (GSTT1 and GSTM1) of glutathione S-transferases.

### The Conundrum of Therapeutic Protection

A conclusion that can be drawn from human studies is that antioxidants are viable therapeutic agents against drug- and noise-induced hearing loss. To what extent dietary manipulation can attenuate presbycusis, however, remains to be established. But even for ototoxicity and noise trauma the situation may not be predictable. Individual genetic predisposition and the physiological (nutritional) state are confounding factors that are capable of influencing the success of therapeutic interventions. Furthermore, subtle forms of auditory damage may escape simple audiometric tests and a final verdict on success or failure of protective strategies may have to await more sophisticated audiological assessments.

There is another unresolved question. While a short-term administration of antioxidants or vitamins might be safe for drug and noise exposure, the hazards of a life-long regimen of nutritional supplements are unknown. Studies in animals as well as clinical trials have warned of a potentially increased risk of cancer and mortality associated with prolonged consumption of antioxidants or vitamins. Specifically, meta-analyses of clinical trials singled out beta-carotene, vitamin A, and vitamin E as associated with increased overall mortality (Miller et al., 2005; Bjelakovic et al., 2014). A well-balanced diet might be all that is needed to maintain health and hearing into old age (Halliwell, 2011).

## Outlook

When we compare the mechanisms of drug, noise, and age-related hearing loss, there appear to be more similarities between drug- and noise-induced hearing loss than between those two and age (Table 1). One of the reasons might be that age-related hearing loss is complexly influenced by genetics and a lifetime of environmental factors that render its mechanisms and manifestations highly variable. For preventive treatments, it appears that supra-normal doses of protective agents are successful to combat the acute damage of noise or drug trauma but generally fail when given over the long term in an attempt to curb the auditory deficiencies in aging.

**Table 1:** **Selected features of acquired hearing loss**.

	Age	Drugs	Noise
***Pathology***			
Organ of Corti	Loss of hair cells in sensorineural hearing loss	Loss of (primarily outer) hair cells	Dislocation of tectorial membrane from stereocilia; breaking of tip links; loss of hair cells
Stria vascularis (and spiral ligament)	Compromised structure and possibly function in some forms of hearing loss	Aminoglycosides: endolymphatic potential (EP) unaffected Cisplatin: EP may be decreased	May reduce EP; loss of fibrocytes
Spiral ganglion and auditory nerve	Reduced in sensorineural hearing loss; loss of synaptic connections	Loss considered mostly secondary; loss of synaptic connections possible	Loss of synaptic connections
***Reactive oxidant species***			
Evidence	Causal relation largely not supported	+	+
Likely sources	Mitochondria	Aminoglycosides: non-enzymatic; mitochondria; NADPH oxidase Cisplatin: mitochondria, NADPH oxidase	mitochondria; NADPH oxidase; calcium influx may cause oxidative stress
***Effects on mitochondria***			
	mtDNA mutations might impair auditory function; specific mutations correlate with presbycusis in human	Aminoglycosides: Bind to mtRNA and impair mitochondrial protein synthesis and function; inhibition of the electron transport chain	Reduced blood flow may impair oxidative phosphorylation
***Potential cell death pathways***			
Apoptosis	+	+	+
Necrosis	+	Aminoglycosides: + Cisplatin: ?	+
MAPK pathway	+	Aminoglycosides: + Cisplatin: JNK ?	+
Caspase-dependent	?	Evidence mostly from *in vitro* studies	+
Caspase-independent	?	*In vivo* +	+
***Potential cell rescue pathways***			
Homeostasis (e.g., Prx3)	+	+	+
NF-κB	Not determined	Aminoglycosides: +	+
PI3-kinase	+	Aminoglycosides: +	?
Heat shock proteins	+	+	+
***Prevention***			
Nutritional supplements	Inconsistent in animals Inconclusive evidence in clinical trials and epidemiological studies	Aminoglycosides: Consistent protection in animals; successful clinical trials. Cisplatin: success in animal studies	Mostly protective in animal studies Inconclusive in clinical or field trials, which are largely of temporary hearing loss

*+, supporting evidence exists; ?, not known or inconclusive. For drug-induced hearing loss, preference was given to results from studies in vivo or with cochlear tissues in vitro; for age-related hearing loss, results from confounded models (See text: mice with Cdh23 mutations or catastrophic mitochondrial mutations) were de-emphasized*.

Looking ahead, regeneration of human hair cells seems decades away but other approaches may provide new directions. Some aspects of ARHI may relate to the phenomenon of cochlear synaptopathy, causing “hidden hearing loss” by the loss of auditory nerve fiber/hair cell connections (Kujawa and Liberman, 2015). In a noise-induced synaptopathy model, the administration and the overexpression of the neurotrophins NT-3 partially rescued the synaptopathy phenotype in mice (Wan et al., 2014). If an appropriate diagnostic method and drug application can be identified for ARHI, the regeneration of the terminal nerve fibers might be a suitable therapeutic option for at least a selected group of patients.

## Conflict of Interest Statement

The authors declare that the research was conducted in the absence of any commercial or financial relationships that could be construed as a potential conflict of interest.
